# Effect of female genital mutilation/cutting; types I and II on sexual function: case-controlled study

**DOI:** 10.1186/s12978-017-0371-9

**Published:** 2017-08-30

**Authors:** Sahar A. Ismail, Ahmad M. Abbas, Dina Habib, Hanan Morsy, Medhat A. Saleh, Mustafa Bahloul

**Affiliations:** 1 Department of Dermatology, Venereology and Andrology, Faculty of Medicine, Assiut University, Assiut, Egypt; 2 Department of Obstetrics and Gynecology, Faculty of Medicine, Assiut University, Assiut, Egypt; 3 Department of Public Health and Community Medicine, Faculty of Medicine, Assiut University, Assiut, Egypt

**Keywords:** Female genital mutilation/cutting (FGM/C), Sexual function, FSFI

## Abstract

**Background:**

The existing literature is contradictory regarding effects of female genital mutilation/cutting (FGM/C) on sexual functions. The aim of this study was to explore the impact of type I and II FGM/C on sexual function of Egyptian women.

**Methods:**

We recruited 197 cut women and 197 control women from those visiting Assiut University hospitals for different reasons. We asked each woman to fill the Arabic female sexual function index (FSFI) (a self reported 19-item questionnaire assessing the main domains of female sexual function). Genital Examination was done to confirm the type of FGM.

**Results:**

Female sexual dysfunction (FSD) was found in 83.8% of FGM/C cases in contrast to 64.5% of the control. The total FSFI score in the FGM/C group (19.82 ± 7.1) was significantly lower than in the control group (23.34 ± 8.1). Concerning the types of FGM/C, type 73.6% of cases had type I and 26.4% had type II. Type I FGM/C was performed mainly by physicians (62.1%) while type II was performed mainly by midwives (44.4%).

FSD was found in 83.4% of FGM/C I cases and in 84.6% of FGM/C II cases. There was no statistically significant difference between the two types of FGM/C as regards total and individual domain scores except for the pain domain. There were significantly lower total and individual domain scores in both FGM/C types except for the desire domain compared to control.

**Conclusion:**

In this study, FGM/C was associated with reduced scores of FSFI on all domains scores, and among both types I and II, both were associated with sexual dysfunction.

## Plain English summary

Female genital mutilation/cutting (FGM/C) is any procedures that cause injury to the female external genitalia without medical causes. It has no religious root and has been condemned by Al-Azhar institution based on the Holy Quraan.

According to the World health organization latest update; over 200 million girls and women alive today have been cut in the Middle East, Africa and Asia.

Its complications are either physical (range from bleeding and infection to death), psychological (such as anxiety and post traumatic stress) or sexual.

The aim of this study was to explore the impact of FGM/C on sexual function of Egyptian women with FGM/C. We recruited 197 cut women and 197 control women from those visiting Assiut University hospitals for different reasons. We asked each woman to fill the Arabic female sexual function index (FSFI) (a self reported 19-item questionnaire evaluating the main domains of female sexual function). Genital Examination was done to confirm the type of FGM/C.

Female sexual dysfunction was found in 83.8% of FGM/C cases in contrast to 64.5% of the control. The total FSFI score in the FGM/C group (19.82 ± 7.1) was significantly lower than in the control group (23.34 ± 8.1). Moreover, the total and domain scores in both types of FGM/C were significantly lower than control except for the desire domain in type I FGM/C.

In conclusion: we have demonstrated that both type I and type II of FGM/C are associated with sexual dysfunction.

## Background

Female genital mutilation/cutting (FGM/C) is a common injurious traditional practice in many countries in Africa, Middle East and other regions around the globe [1]. It was defined by the World Health Organization (WHO) as “all procedures that lead to partial or complete excision of the external female genitalia or other forms of injury to the female genital organs for non-medical causes” [2].

According to the 2014 Egypt Demographic and Health Survey (EDHS), and in spite of the total ban of this practice by the government, the prevalence of FGM/C in married women between the ages of 15 and 49 was 92% [3].This harmful practice is usually a trial to control girl’s sexual life and decrease their sexual desire to encourage innocence, virginity and fidelity [4].

WHO distinguishes four types of FGM/C [2]. Type I: Clitoridectomy; partial or complete excision of the clitoris and/or the prepuce. Type II: Excision; partial or complete removal of the clitoris and labia minora, with or without removal of labia majora. Type III: Infibulation; reduction of the vaginal orifice with a seal formed by cutting and repositioning of labia minora and/or labia majora, with or without removal of the clitoris. Type IV: All other harmful procedures to the genitalia such as pricking, piercing, incising, scraping and cauterization. In Egypt types I and II are the most frequently used methods while Types III and IV are fairly rare [3].

FGM/C is always traumatic and has no identified health benefits [2, 5].Its complications are either physical (range from bleeding and infection to death), psychological (such as anxiety and post traumatic stress) or sexual [6, 7].

The existing literature is unclear what the effects FGM/C have on sexual function and desire [8–11]. Several African studies have defied the negative effect of FGM/C on sexual functions. However, there is increasing evidence that FGM/C damages sexual function which would appear logical after the damage of sexually sensitive organ such as the clitoris [12]. Berg and Denison conducted a meta-analysis of the sexual consequences of FGM/C, combining total of15 studies from seven countries [13]. They reported heterogeneity of the available studies and varying methodological quality.

The aim of this study was to explore the impact of FGM/C types I and II on sexual function of Egyptian women who have undergone this procedure.

## Methods

This case-control study had been conducted at the outpatient clinics of Dermatology and Gynecology & Obstetrics departments at Assiut University Hospitals (tertiary referral university hospital) from December 2015 to June 2016. After the objective of the study had been explained, all women gave their written informed consent to participate in the study which was approved by the institutional review board.

Sample size was calculated by Open Epi Info [14] for case control study design with two sided confidence interval 95%, power 80%, ratio of controls to cases 1, controls exposure 55%, cases exposure 75% [15–18].and odds ratio 2.45. The calculated sample size by Fleiss with continuity correction (CC) is 99 for cases and equal number 99 for control with total number 198. We doubled the sample to get more informative data and increase the power of the study.

The study included 394 healthy sexually active Egyptian women (197 with FGM/C and 197 without FGM/C) who had visited the hospital either for routine check-up, for mild dermatological illness or accompanying other patients. Women with chronic medical illnesses, psychiatric illness, pregnancy and lactation, illiterate women and those with no sexual activity in the last 6 months were excluded from the study.

Women were interviewed in a private room with their names and addresses not recorded to ensure confidentiality. They were asked to fill out the Arabic version of the Female Sexual Function Index (FSFI) by themselves [19].This 19-item standardized questionnaire covers six domains; desire, arousal, lubrication, orgasm, satisfaction, and pain. It evaluates sexual function during the last month. The cut-off score to define sexual dysfunction on the total FSFI score is 28.1. One of the female investigators was available at the interview room in case women need to clarify anything and to make sure all items were answered. Genital examination of all participants was performed after the questionnaire had been completed to confirm the type of FGM/C.

### Statistical analysis

All data were analyzed using SPSS software (Chicago, IL, USA) version 21. Comparison between categorical variables in both groups was done by χ2 test, and continuous variables were compared using Student’s t test. For statistical analysis, we tested the different scores for normality by Shapiro–Wilks test, and they were normally distributed, so they are presented as mean ± SD and compared with the Student’s t test. We considered *p* value <0.05 as a significant value.

## Results

Out of 245 invited women with FGM/C, 197 agreed to participate in the study (80.4%). We invited 233 non cut women to the control group until equal number of women (197) agreed to participate (84.5%).

There were no statistically significant differences between the FGM/C and the control groups as regards age, educational level, residence and parity. Within the FGM/C group the mean age was 32.68 ± 10.35 years (range 18–43) compared to 31.71 ± 8.58 (range 19–43) years in the control group. High education was reported in 55.6 and 58.5% of cases and control respectively, 58.6% of cases had rural residence versus 51.4% of the control and the mean number of children in the FGM/C and control women were 2.01 ± 1.48 (range 0–6) and 1.75 ± 1.36 (range 0–5) respectively.

**Fig. 1:**
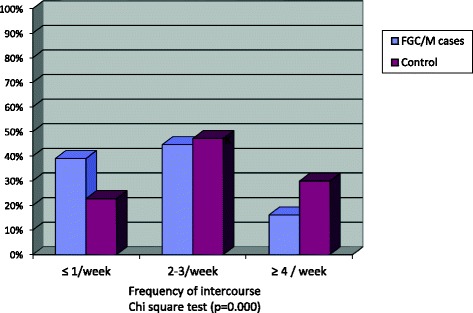
Frequency of sexual intercourse in cases of FGM/C compared to control

Figure 1 illustrates that in 39% of FGM/C cases the frequency of sexual intercourse was ≤1time/week compared to 22.8% of the control and in only 16.2% of cases the frequency was ≥4 times/ week compared to 29.9% of the control (*p* = 0.000).

Concerning the types of FGM/C, 73.6% (*n* = 145) of cases had FGM/C type I and 26.4% (*n* = 52) had FGM/C type II. The mean age at the procedure was 7.13 ± 2.16 years, 49.7% of cases were performed by physicians and 68.5% were performed at home (Table 1).

**Table 1:** Characters of female genital mutilation/cutting

Variable	Total FGM/C (*n* = 197)	FGM/C I (*n* = 145)	FGM/C II (*n* = 52)	*p*-value FGM/C I vs FGM/C II
Age at FGM/C: mean ± SD	7.13 ± 2.16	7.28 ± 2.27	6.72 ± 1.78	0.065
Performer of FGM/C: n (%)				
Barber	14 (7.1)	5 (3.4)	9 (17.3)	0.000^a^
Midwife	63 (32.0)	35 (24.1)	28 (44.4)	
Nurse	22 (11.2)	15 (10.3)	7 (13.5)	
Physician	98 (49.7	90 (62.1)	8 (15.4)	
Place of FGM/C: n (%)				
Home	135 (68.5)	91 (62.8)	44 (84.6)	0.002^a^
Health related facility	62 (31.5)	54 (37.2	8 (15.4)	

*FGM/C* female genital mutilation/cutting

^a^statistically significant difference by Chi Square test (Student t-test was used to compare mean ages)

Comparing the 2 types of FGM/C, it was revealed that there was no significant difference as regard the mean age of the procedure (*p* = .0.065). While type I FGM/C was performed mainly by physicians (62.1%), type II FGM/C was performed mainly by midwives (44.4%) (*p* = 0.000). Type I FGM/C constitute the majority of cases performed at health related facility (54 out of 62 cases) (*p* = 0.002).

Regarding sexual function, female sexual dysfunction (FSD) was found in 83.8% of FGM/C cases in contrast to 64.5% of the control. The total FSFI score in the FGM/C group (19.82 ± 7.1) was significantly lower (*p* = 0.000) than in the control group (23.34 ± 8.1). Moreover, the domain scores in FGM/C group were significantly lower than control in all domains namely desire, arousal, lubrication, orgasm, satisfaction, and pain (Table 2).

**Table 2:** Female sexual function index total and domain scores in the study groups

Variable	FGM/C (*n* = 197)	Control (*n* = 197)	*P*-value
Desire	3.44 ± 1.4	3.72 ± 1.1	0.028^a^
Arousal	3.12 ± 1.64	3.67 ± 1.72	0.001^a^
Lubrication	3.54 ± 1.68	4.12 ± 1.74	0.001^a^
Orgasm	3.24 ± 1.54	3.91 ± 1.78	0.000^a^
Satisfaction	3.66 ± 1.45	4.02 ± 1.57	0.002^a^
Pain	3.0 ± 1.52	3.89 ± 1.6	0.000^a^
Total score	19.82 ± 7.1	23.34 ± 8.1	0.000^a^

*FGM/C* female genital mutilation/cutting

^a^statistically significant difference by Student t-test

FSD was found in 83.4% of FGM/C I cases and in 84.6% of FGM/C II cases. There was no statistically significant difference between the two types of FGM/C as regards total and individual domain scores except for the pain domain (Table 3). Comparing the two types of FGM/C with the control revealed significant lower total and domain scores in both FGM/C types except for the desire domain which showed no statistically significant difference compared to control (*p* = 0.141).

**Table 3:** The Female sexual function index scores in the study groups

Variables	FGM/C I (*n* = 145)	FGM/C II (*n* = 52)	Control (*n* = 197)	*P*-value	*P*-value^a^	*P*-value^b^	*P*-value^c^
Desire	3.51 ± 1.43	3.25 ± 1.31	3.72 ± 1.1	0.038^d^	0.229	0.141	0.020^d^
Arousal	3.15 ± 1.59	3.02 ± 1.77	3.67 ± 1.72	0.005^d^	0.628	0.005^d^	0.021^d^
Lubrication	3.62 ± 1.58	3.32 ± 1.62	4.12 ± 1.74	0.002^d^	0.247	0.006^d^	0.002^d^
Orgasm	3.34 ± 1.54	2.99 ± 1.55	3.91 ± 1.78	0.000^d^	0.166	0.002^d^	0.000^d^
Satisfaction	3.61 ± 1.45	3.43 ± 1.45	4.02 ± 1.57	0.008^d^	0.458	0.011^d^	0.012^d^
Pain	3.16 ± 1.53	2.64 ± 1.36	3.89 ± 1.6	0.000^d^	0.024^d^	0.000^d^	0.000^d^
Total score	20.26 ± 6.9	18.64 ± 7.5	23.34 ± 8.1	0.000^d^	0.177	0.000^d^	0.000^d^

*FGM/C* female genital mutilation/cutting

*P*-value: difference between the 3 groups by ANOVA test

*P*-value ^a^difference between FGM/C I and FGM/C II groups by Student t- test

*P*-value ^b^difference between FGM/C I and control groups by Student t- test

*P*-value ^c^difference between FGM/C II and control groups by Student t- test

^d^statistically significant

## Discussion

FGM/C is recognized worldwide as a violation of the girls’ and women’s human rights and constitutes an intense form of discrimination against them due to the severe medical risks and health consequences. While the exact number of girls and women exposed to FGM/C is undetermined, it is believed that more than 200 million women have been subjected to FGM/C, more than half of them were in Indonesia, Egypt and Ethiopia [2].

In some Muslim countries including Egypt where FGM/C is common it is often wrongly alleged that the basis for performing FGM/C is religious instruction [11]. Comprehensive discussion of the Islamic viewpoints on this subject is beyond the scope of this article however, it is sufficient to report that the practice of FGM predates Islamic times (and also Judaism) and is common in religious and nonreligious groups [20].

The current study revealed a significant association between FGM/C and decline in the female sexual functions with significant difference between cases and control in the total and individual FSFI domain scores. This decline is also manifested by the decreased frequency of sexual intercourse in cases compared to control. These findings are consistent with previous studies evaluating the relation between FGM/C and female sexual function. In a recent study by Mahmoud, 2016 in Egypt, The total score of FSFI for cases was 14.3 ± 5.93 versus 25.9 ± 3.44 for control (*P* = 0.000) [4]. Biglu et al., 2016 also proved that the total scores for circumcised women was significantly lower than control women (17.9 ± 5.39 versus 25.3 ± 4.34 respectively, *p* = 0.000) [11].

In the study of Alsibiani and Rouzi on 260 women in Saudi Arabia, no difference in the mean desire or pain score was observed. While, there were statistically significant differences in the arousal, lubrication, orgasm, and satisfaction, as well as the overall sexual function score between circumcised and control women [8].

On the other hand in a study by Catania et al. [9], 57 women with type III FGM/C reported higher scores than controls in several FSFI domains, but this study was confounded by the unmatched control group consisting mainly of western women. In this case–control study, we tried to eliminate this confounder by recruiting control women from equivalent cultural backgrounds with matching residence and education.

Our findings demonstrated that both type I and type II FGM/C are associated with sexual dysfunction. The mean total and individual domain scores of FSFI in women with both FGM/C types were significantly lower than the control group except the desire score in FGM/C type I.

On the contrary, Thabet and Thabet [21] studied 147 Egyptian women and found that women with type I FGM/C had no reduction in sexual function, whereas those who had undergone type II or type III circumcision had several sexual problems. Another study by Andersson et al. reported that sexually active women who had undergone FGM/C type III differed significantly from sexually active controls in their SQOL-F score and not types I and II [7].

Traditionally, FGM/C was done by midwives, but the practice is increasingly medicalized and more health-care providers are performing the procedure [22, 23]. This is supported by our results as nearly half of FGM/C cases (49.7%) were performed by physicians.

Medicalization of FGM/C is proposed by some health professionals to reduce the incidence of its complications due to the alleged notion that it will result in milder forms of FGM/C. This assumption was supported by our findings that type I FGM/C was performed mainly by physicians (62.1%) and type II FGM/C was performed mainly by midwives (44.4%), however both types of FGM/C had a proven negative effect on women sexuality. Therefore, medicalization of FGM/C violates the code of medical ethics and research has shown that it will not reduce the long term complications of FGM/C. Moreover it would hinder the global efforts to eradicate this harmful practice [20].

Also, most of FGM/C cases (68.5%) were carried out at home which may raise the possibilities of infection and scar formation that may affect the future sexual functions of those girls. This may explain the pain domain defect in both types of FGM/C reported in our study and the significant lower pain score in type II compared to type I. These results are in line with several previous reports that dyspareunia is more prevalent in the women with FGM/C compared the women without it [13].

The main strength of the current study is confirming the type of FGM/C by meticulous genital examination, as self reporting of FGM/C and its different forms (used by many previous studies) is unreliable and both underreporting and over reporting have been documented [24]. Other strength points include the use of a standardized questionnaire that has been validated for the Egyptian population [19], and inclusion of a good number of cases and their matching control as regards age, residence, education and parity. However, multiple centers’ study will better address the problem as it is difficult to generalize from small one center study. It is worth noting that recruiting of controls in this study was very difficult due to the high prevalence of FGM/C so that suitable matching controls were not easy to find.

A limitation was that the subject of FGM/C is very sensitive and debatable in our conservative community, especially when it comes to its effect on sexual function. Some women may be reluctant to complain about the discomforts they have or to convey their FGM/C in negative terms as it might mean having negative feelings about their parents being responsible for the FGM/C. Also it might mean their condemnation of the religious orders. Others tend to over report their FGM/C negative health consequences as a way to pronounce their frustration from this physical and psychological assault.

Efforts by religious and medical authorities to demonstrate that all types of FGM/C including type I are associated with a long list of health consequences including sexual dysfunction, will help toward abandoning the procedure.

## Conclusions

In this study, FGM/C is associated with reduced scores of FSFI on all domain scores. We have also demonstrated that both type I and type II of FGM/C are associated with sexual dysfunction. Although it is difficult to withdraw generalized conclusions, the findings of this brain storming study draw attention to a very sensitive issue.

## Abbreviations

EDHS: Egypt demographic and health survey
FGM/C: Female genital mutilation/cutting
FSD: Female sexual dysfunction
FSFI: Female sexual function index
SD: Standard deviation
WHO: World Health Organization
χ^2^: Chi squared test
